# RalGPS2 Is Essential for Survival and Cell Cycle Progression of Lung Cancer Cells Independently of Its Established Substrates Ral GTPases

**DOI:** 10.1371/journal.pone.0154840

**Published:** 2016-05-05

**Authors:** Adriana O. Santos, Maria Carla Parrini, Jacques Camonis

**Affiliations:** 1 INSERM U830, Institut Curie, PSL Research University, Paris, France; 2 The Biophenics Project, Translational Research Department, Institut Curie, Paris, France; Texas A&M University Health Science Center, UNITED STATES

## Abstract

The human genome contains six genes coding for proteins validated *in vitro* as specific activators of the small GTPases “Ras-related protein Ral-A” and “Ras-related protein Ral-B”, generically named Ral-guanine nucleotide exchange factors (RalGEF). Ral proteins are important contributors to Ras oncogenic signaling, and *RAS* oncogenes are important in human Non-Small Cell Lung Carcinoma (NSCLC). Therefore in this work, RalGEF contribution to oncogenic and non-oncogenic features of human NSCLC cell lines, as anchorage-dependent and independent growth, cell survival, and proliferation, was investigated. Among all human RalGEF, silencing of *RGL1* and *RALGPS1* had no detectable effect. However, silencing of either *RGL2*, *RGL3*, *RALGDS* or, to a larger extent, *RALGPS2* inhibited cell population growth in anchorage dependent and independent conditions (up to 90 and 80%, respectively). *RALGPS2* silencing also caused an increase in the number of apoptotic cells, up to 45% of the cell population in transformed bronchial BZR cells. In H1299 and A549, two NSCLC cell lines, *RALGPS2* silencing caused an arrest of cells in the G0/G1-phase of cell cycle. Furthermore, it was associated with the modulation of important cell cycle regulators: the E3 Ubiquitin Protein Ligase S-phase kinase-associated protein 2 (Skp2) was strongly down-regulated (both at mRNA and protein levels), and its targets, the cell cycle inhibitors p27 and p21, were up-regulated. These molecular effects were not mimicked by silencing *RALA*, *RALB*, or both. However, *RALB* silencing caused a modest inhibition of cell cycle progression, which in H1299 cells was associated with Cyclin D1 regulation. In conclusion, *RALGPS2* is implicated in the control of cell cycle progression and survival in the *in vitro* growth of NSCLC cell lines. This function is largely independent of Ral GTPases and associated with modulation of Skp2, p27 and p21 cell cycle regulators.

## Introduction

Ras proteins are small GTPases frequently mutated in human cancer. They have many downstream effectors, including the small GTPases “Ras-related protein Ral-A” (RalA) and “Ras-related protein Ral-B” (RalB), which are activated by Guanine nucleotide Exchange Factors (RalGEF). The RalGEF-Ral pathway gained special attention after the finding that the expression of a mutant form of the GTPase HRas that specifically and exclusively activates this signaling pathway is sufficient for Ras-induced transformation of human cells [1]. There are six Ral-specific guanine nucleotide exchange factors. Four of them, the Ral guanine nucleotide dissociation stimulator (RalGDS), the Ral guanine nucleotide dissociation stimulators-like 1, -like 2 and -like 3 (RalGDS-like 1, -like 2 and -like 3 or alternatively RGL1, RGL2 and RGL3), harbor Ras-binding domains and can therefore directly signal downstream the Ras proto-oncogenes toward the Ral GTPases. In addition, the Ral guanine nucleotide exchange factor with PH domain and SH3 domain-binding motif 1 (RalGPS1) and Ral guanine nucleotide exchange factor with PH domain and SH3 domain-binding motif 2 (RalGPS2) are two Ras-independent RalGEF [2]. Ras-dependent RalGEF (reviewed in [3]) have been more studied than the Ras-independent RalGEF, which known functions are limited to cytokinesis of HeLa cells [4] and rat pheochromocytoma differentiation under Nerve Growth Factor stimulus [5]. Additionally, despite extensive work on RalA and RalB GTPases contribution to human cancer [6], only recently their role in lung cancer, frequently harboring Ras oncogenic mutations, has been reported [7,8]. Nevertheless, RalGEF role in human Non-Small Cell Lung Carcinoma (NSCLC) remains unknown.

In this work, the contribution of the six RalGEF genes to human NSCLC cell survival, proliferation, and transformed features was investigated. The main strategy was to systematically silence each RalGEF in NSCLC cell lines bearing different Ras mutations (Table 1) and to study the functional contributions of each RalGEF gene. In this way, we were able to uncover unsuspected functions of a particular RalGEF, the RalGPS2 protein in cell survival and G1-S cell cycle phase transition.

**Table 1:** Histology and Ras mutation type of the cell lines used in this work.

Cell line	Tissue Histology	Ras mutation
A549	Human lung carcinoma	KRas^**G12S**^
H23	Human lung adenocarcinoma	KRas^**G12C**^
H1299	Human lung carcinoma	NRas^**Q61K**^
H838	Human lung adenocarcinoma	WT
HeLa	Human cervix carcinoma	WT
HekHT	Immortalized human embryonal kidney	WT
HekRasV12	HRas^G12V^-transformed human embryonal kidney	HRas^G12V^
BEAS-2B	Immortalized human bronchial	WT
BZR	HRas^G12V^-transformed human bronchial	HRas^G12V^

## Materials and Methods

### Cell lines and culture

HeLa and the human NSCLC cell lines H23, H1299, A549, and H838, were from the American Type Culture Collection (ATCC, catalog numbers CCL-2, CRL-5800, CRL-5803, CCL-185, CRL-5844, respectively) and were grown according to supplier recommendations. The human HeLa cell line was cultured in Dulbecco’s modified Eagle medium (DMEM) (GIBCO, ref. 41966–029 Invitrogen) supplemented with 10% heat-inactivated FBS, and 2 mM L-Glutamine. NSCLC cell lines were cultured in RPMI-Glutamax (GIBCO, ref. 61870–010, Invitrogen) supplemented with 10% heat-inactivated fetal bovine serum (FBS, MP Biomedicals, ref. 092910154), 4.5 g/l glucose (ref. G8769, Sigma) and 1 mM sodium pyruvate (ref.15070-063, Invitrogen). All cells were maintained in exponential growth conditions at 37°C in a humidified atmosphere (90%), containing 5% CO_2_. Routinely, no antibiotics were added to culture medium and cultures were confirmed to be free from mycoplasma contamination by Polymerase chain reaction (PCR) (VenorGeM Classic, ref. 11–1100).

Human embryonic kidney cells stably expressing the early region of SV40, the catalytic subunit of telomerase hTERT HEK-HT (HekHT) and HEK-HT-Ras^G12V^ (HekRasV12) cells were kindly provided by Christopher Counter [9,10] and were cultured in DMEM supplemented with 10% heat-inactivated FBS, 2 mM L-Glutamine, and antibiotics of selection (hygromycin 100 μg/ml, neomycin 400 μg/ml, and in the case of HekRasV12 also puromycin 0.5 μg/ml).

BEAS-2B and BZR were purchased from the ATCC (catalog numbers CRL-9609 and CRL-9483, respectively) and were cultured in LHC-9 medium (GIBCO, ref. 12680–013, Invitrogen) in coated plates (without any added BSA). The coating solution was composed of 0.01 mg/ml Fibronectin (SIGMA, ref. F0895, Sigma-Aldrich) and 0.03 mg/ml Collagen Type I (ref. 207050357, Institut de Biotech., France) in LHC Basal medium with 0.01 mg/ml BSA (Sigma). For splitting cells, or plating for experiments, cells were detached using Accutase Solution (SIGMA, ref. A6964, Sigma-Aldrich), centrifuged and resuspended in fresh medium.

### siRNA oligonucleotides and transfection of cells

SiRNA were mostly purchased from Eurogentec S.A (Belgium) in the form of desalted annealed duplexes, either dry or in solution. The control siRNA sequence (ON-TARGETplus Non-targeting siRNA #1, named siNT in the abbreviated form) was always purchased from Dharmacon, (Thermo Fisher Scientific, USA). Target sequences are described in Table 2. -siRNA stock concentrations were was always confirmed by measuring solution absorbance at 260 nm, using the extinction coefficient as calculated by Dharmacon’s website for each siRNA hybrid (http://www.thermoscientificbio.com/custom-modified-sirna/), diluted in siRNA buffer (300 mM KCl, 30 mM HEPES pH 7.5, 1.0 mM MgCl_2_ –from 5x buffer solution purchased from Dharmacon, USA) and aliquoted to minimize repeated freezing/thawing cycles. In some experiments pools of siRNAs were used, which consisted in mixtures of equimolar amounts of 3 oligonucleotides.

**Table 2:** SiRNA target sequences.

siRNA name	Target (sense) sequence (no overhangs*)	position / mRNA sequence ID	Origin
**siLuc**	5'-CGUACGCGGAAUACUUCGA-3'	*not applicable*	Eurogentec
**siNT**	ON-TARGETplus Non-targeting siRNA #1	*not applicable*	Dharmacon
**siPLK1**	5'-CGGGCAAGAUUGUGCCUAA-3'	292–310 / NM_005030.3	Eurogentec
**siRalA_I**	5'-GAGACAACUACUUCCGAAG-3'	546–564 / NM_005402.3	Eurogentec
**siRalA_II**	5'-GACAGGUUUCUGUAGAAGA-3'	714–732 / NM_005402.3	Eurogentec
**siRalB_107**	5'-UGACGAGUUUGUAGAAGAC-3'	298–316 / NM_002881.2	Eurogentec
**siRalB_utr**	5'-GAGCCCAGUAUUCACAUUU-3'	2051–2069 / NM_002881.2	Eurogentec
**siRalB_1749**	5'-CAAAGACGUGAUGAGUUAA-3'	1749–1767 / NM_002881.2	Eurogentec
**siRGL1_I**	5'-CAGUAUCAUUGUUAAGUGA-3'	4190–4202 / NM_015149.4	Eurogentec
**siRGL1_II**	5'-CCAUAAUACAGCUCCUAAA-3'	4440–4458 / NM_015149.4	Eurogentec
**siRGL2_2272**	5'-GCUAAUGUAUUCUACGCCA-3'	2703–2721 / NM_004761.4	Eurogentec
**siRGL2_2296**	5'-GGAUGGAGCUUCACACGAU-3'	2723–2741 / NM_004761.4	Eurogentec
**siRGL2_2333**	5'-CGAAGGUCCUCUACUGCUA-3'	2760–2778 / NM_004761.4	Eurogentec
**siRGL3_I**	5'-ACACAGCCCUGCCGGAUAU-3'	1368–1386 / NM_001161616.1	Eurogentec
**siRGL3_II**	5'-GCGUCAGCAUCGACAAUGA-3'	1935–1953 / NM_001161616.1	Eurogentec
**siRalGDS_1365**	5'-CGGGAACCGAAGCACGAAA-3'	1367–1385 / NM_006266.2	Eurogentec
**siRalGDS_ft9**	5'-AGCAAAUGCUAGACUUGAA-3'	3585–3603 / NM_006266.2	Qiagen, Eurogentec
**siRalGDS_utr**	5'-AACCAGAGGACUAGCUGACUU-3'	2986–3006 / NM_006266.2	Eurogentec
**siRalGPS1_ups1**	5'-GAACAAAGAUCCAAUCAGA-3'	946–964 / NM_014636.2	Eurogentec
**siRalGPS1_ups3**	5'-GGAUAUACCUGUGUUUAAA-3'	450–468 / NM_014636.2	Eurogentec
**siRalGPS2 #231**	5'-GAUUCAGCAUACCCAUCAA-3'	975–993 / NM_152663.3	Eurogentec
**siRalGPS2 #232**	5'-GCGGGUCAGAUAACAUUAAT-3'	498–517 / NM_152663.3	Eurogentec
**siRalGPS2_ft10**	5'-CAGUCGUUGGAGUUCUCAA-3'	2205–2222 / NM_152663.3	Qiagen, Eurogentec

* Extra two-nucleotide overhangs were synthesized as deoxyribonucleotides (either dTdT or complementary to mRNA in the antisense strand)

The siRNA oligonucleotides were added to cells in the form of 5X lipoplexes prepared in OPTIMEM (Invitrogen) with a commercial transfection reagent (Lipofectamine RNAiMAX, ref. 13778030, Invitrogen), achieving the final concentration optimized for each cell line (usually 10 nM or 12 nM). The ratio of transfection reagent to siRNA was either 1 μl:10 pmol or 1 μl:12 pmol, in all other aspects prepared according to the manufacturer’s instructions. Experimental factors were optimized for each cell line in order to have minimum transfection toxicity (difference between untreated and siNT-treated cells) and maximum positive control effect (siPLK1).

### Resazurin reduction assay

Cells were plated the evening before transfection in 96 well-plates, at the optimized density for each cell line that guaranteed under-confluence at the final time point. Untreated, siNT and siPLK1 controls were present at each plate. Optimal conditions were those where in average the relative resazurin reduction of cells treated with siNT (Non-Targeting control sequence) was above 75%, and of cells treated with siPLK1 was 10% or less. For transfection, medium was replaced by 64 μl of fresh complete medium without antibiotics and siRNA:Lipofectamine RNAiMax lipoplexes were added in 16 μl, to wells in quadruplicate. Fresh medium was added to wells at 24 or 48 h post transfection. At the final time point (72 h for Hela, 96h for A549, H1299, HekHT and HekRasV12, and 120h for H23 cells) medium was replaced by 200 μl RPMI without phenol red, containing resazurin (SIGMA^®^, Sigma-Aldrich Chemie GmbH) at 10 μg/ml and incubated at 37°C for 2 h-4 h. Fluorescence was read with a plate reader at the wave lengths of 540 nm (excitation) and 620 nm (emission), and results were expressed as percentage of the fluorescence signal of untreated cells after background subtraction.

### Western blots

Cells were plated in 6-well plates over-night and before transfection the medium was replaced by 2 ml of fresh complete growth medium (without antibiotics). Cells were treated with 0.5 ml of 5X siRNA lipoplexes as described in the previous section. 48 h to 96 h post transfection cells were washed with phosphate buffer saline without calcium and lysed with Triton lysis buffer (1% Triton X-100, 5 mM MgCl_2_, 150 mM NaCl, 50 mM Tris, pH 7.4) supplemented with protease inhibitors cocktail tablet (ref. 11873580001 Roche) according to standard procedures, or directly lysed with 2X Laemmli Buffer (Tris HCl 62.5 mM, glycerol 15%, SDS 4%, bromophenol blue 0.01%, DTT 200 mM, pH 6.8) supplemented with phosphatase inhibitors and boiled. Soluble protein samples from Triton lysates were quantified using BCA protein assay kit (Pierce, ref. 23225, Thermo Scientific), then diluted in lysis buffer to the same concentration among tubes, Laemmli buffer added to 1X concentration, and boiled. Laemmli buffer lysates were quantified using a turbidimetric Protein Quantification Assay (ref. 740967.250, Machery-Nagel) and diluted to equivalent concentrations. Samples were loaded onto precast (4–15% or “any kD” gradient polyacrylamide gels, Bio-Rad) or onto freshly cast 10 or 12% polyacrylamide gels. After electrophoresis, the proteins were transferred to a 0.2 μM nitrocellulose transfer membrane (Whatman). The following primary antibodies and dilutions were used: mouse anti-Adaptin α (ref. 610502, BD Biosciences, 1:1000), mouse anti-RalA (ref. 610222, BD Biosciences, 1:1000), mouse anti-Actin (ref. A5441, Sigma-Aldrich, 1:10000), rabbit anti-RalB (ref. 3523, Cell Signaling, 1:500), mouse anti cleaved PARP (ref. 552597, BD Pharmingen, 1:1000), rabbit anti-Cyclin D1 (ref. 2922, Cell Signalling, 1:1000), mouse anti-Cyclin D3 (ref. 2936, Cell Signalling, 1:2000), mouse anti-p21 (ref. 2946, Cell Signalling, 1:1000), rabbit anti-p27 (ref. sc-527, Santa Cruz, 1:1000), rabbit anti-Skp2 (ref. sc-7164, Santa Cruz, 1:1000). Appropriate conjugated secondary antibodies were used either for visualization of blots by enhanced chemiluminescence detection (Western Lightning^®^ Plus-ECL, PerkinElmer) or LICOR Odyssey Infrared Imaging System (LI-COR Biosciences).

### Quantitative real-time reverse-transcription PCR

Cells were plated the evening before transfection and transfected as described for Western blot analysis. At the indicated time, supernatant was removed and cells were lysed with 350 μl of RTL buffer from RNeasy^®^ mini kit (QIAGEN GmbH, Hildon, Germany) with extemporaneously added beta-mercaptoethanol, and kept at -80°C. Total RNA was isolated with the RNeasy^®^ mini kit, according to the manufacturer’s instructions, including genomic DNA elimination. The absorbance at 260 nm, and 260/230–260/280 nm ratios, were used to quantify and determine the quality of the isolated RNA. 1 μg of RNA was reverse transcribed using the iScript^™^ cDNA Synthesis Kit (Bio-Rad Laboratories) according to the manufacturer’s protocol. Quantitative reverse-transcription real-time PCR (qPCR) was performed using either specific primers (purchased at Sigma Proligo, Table 3) and SYBR Green PCR Master Mix (ref. 4367659, Applied Biosystems), or Taqman probe sets (Applied Biosystems, Table 4) and Taqman PCR Master Mix (Applied Biosystems). Reactions were performed in 25 μl (SYBR green) or 20 μl (Taqman) of final volume using the Chromo4 Thermal Cycler (Bio-Rad Laboratories). The annealing and extension temperature was 60°C and primers were at 300 nM. In SYBR Green assays the melting curve protocol was started immediately after amplification to confirm the specificity of the reaction. The percentage of each mRNA was calculated by the Pfaffl method [11] using *Beta-2-Microglobulin* as reference gene. Primer efficiency was experimentally determined from calibration curves included at least in the first three reactions, and an average efficiency value was used the other times.

**Table 3:** SYBR Green qPCR primers.

Gene name	Sense, Antisense	Source
B2M*	5’- GAGTATGCCTGCCGTGTG-3’, 5’-AATCCAAATGCGGCATCT-3’	Sigma Proligo
*RGL1*	5’-TGGTGATCAGGAATGCAATCG-3’, 5’-CGGCATCATCCGTGTGAGATA-3’	Sigma Proligo
*RGL3*	5’-CCTTGCAGAAGCACAATGTGC-3’, 5’-CGTTGGCATTGTCAGGAATCA-3’	Sigma Proligo
*RALGDS*	5’-GTGCTCCTTGCCCTTCTTGT-3’, 5’-CATGGCCTTGCGGATTACAG-3’	Sigma Proligo
*RALGPS1*	Hs_RALGPS1_1_SG Cat no QT00054383	QuantiTect Primer Assay_QIAGEN

*Beta-2-Microglobulin

**Table 4:** Taqman qPCR probe sets.

Gene name	Probe set code	Source
B2M*	Hs00984230_m1	Applied Biosystems
*RGL2*	Hs00191084_m1	Applied Biosystems
*RALGPS2*	Hs00216096_m1	Applied Biosystems

*Beta-2-Microglobulin

### Trypan blue exclusion assay

Cells were plated in 6-well plates and transfected with siRNAs as described above. At each 24 h interval, both supernatants and cells were detached with trypsin and collected in conic tubes, centrifuged, then cells were resuspended in approximately 0.7 ml of fresh medium. Viable and unviable cells were automatically counted using Vi-Cell cell counter (Backman) which determines cell viability by trypan blue exclusion.

### Anchorage independent growth

H1299 cells were plated in 6-well plates and transfected with siRNA as described above. The following day (about 20 h later), non-adhering 6-well plates (untreated plastic further coated with 0.01% pluronic, Sigma-Aldrich), were coated with 2 ml 0.75% low melting temperature agarose (Sigma-Aldrich), prepared by mixing equal volumes of 1.5% agarose prepared in water and 2X complete medium without phenol red, and left to solidify at room temperature. Before use, extra low melting temperature agarose at 0.75% was prepared and kept in a water bath at 37°C. Cells were collected with Accutase, counted, diluted to the same concentration (25000/ml) and distributed in 5 ml polypropylene tubes (0.8 ml per tube). An aliquot of the same cell suspension was distributed in parallel to replicate wells of white 96-well plates to confirm input by Celltiter Glo assay (ref. G7571, Promega). 3.2 ml of 0.75% agarose was thoroughly but carefully mixed with cells and 1 ml/well was immediately plated in triplicate. After solidification of agarose, extra medium (without phenol red) was added to wells, plates were transferred to the cell incubator and liquid medium on the top carefully replaced every 2–3 days.

After 14 days, medium was removed, and 0.3 ml of a 0.05% MTT solution in PBS was added to each well. Plates were incubated for 30 min to 1 h at 37°C and then scanned at high resolution (Epson Perfection V700 Photo) and colonies counted using the software ImageJ.

### Cell cycle distribution and active Caspase-3 evaluation by flow cytometry

Cells were plated in 12-well plates and transfected with siRNAs as described above. At 72 h post-transfection, supernatants were collected in 15 ml tubes and put on ice. Cells were harvested with Accutase and added to each respective supernatant tube. Tubes were centrifuged at 4°C, and cell pellets were resuspended in PBS containing 0.05% BSA. Cells were fixed and permeabilized by adding pre-cooled -20°C 70% ethanol drop-wise to cells, then kept in ice for at least 30 min. Cells were washed with PBS, then with PBS containing 0.05% BSA, and resuspended in 0.25 to 0.4 ml PBS containing 0.05% BSA plus propidium iodide (50 μg/mL). RNase (5 μl of a stock concentration of 10 mg/ml) and 6 μl of anti-active Caspase-3 antibody (Ref 559341, BD Biosciences) were added to 0.2 ml of propidium iodide-stained cells in 5 ml FACS tubes, incubated protected from light at room temperature for a minimum of about 15 min and analyzed by flow cytometry (BD™ LSRII, BD Biosciences), or kept at 4°C and acquired latter the same day. Data analyses were performed in the case of Active Caspase-3 with FlowJo Software (FloJo, LLC) or, in case of cell cycle distribution, with ModFit LT™ (Verity Software house).

### Ral-GTP Pull-down

Cells were plated in 15 cm plates, and harvested while the cell monolayer was less than 70–80% confluent. One plate each time, in cold room, on ice, cells were washed once with ice cold PBS. Cells were lysed with Lysis buffer (1 ml, Tris.HCl 50 mM, pH 7.4, NP40 1%, Glycerol 15%, NaCl 200 mM, MgCl2 5 mM, supplemented with protease inhibitors) and lysates were cleared by centrifugation (2 min at 10000 rpm, in cold room). The supernatants were aliquoted in 3 tubes (for protein quantification, input Western blots and pull-down experiments), then immediately snap frozen in liquid nitrogen.

Glutathion magnetic beads (Pierce #88822) were washed 3 times with 3-fold their volume of washing buffer A (Tris.HCl 125 mM, pH 8, NaCl 150 mM, supplemented before use with protease inhibitors), incubated 30 min to 1 h in the same buffer on a rotating wheel at 4°C with lysates from *E*. *coli* cells expressing GST-fused Ral Binding Domain from Sec5 (GST-Sec5-RBD, RBD being the N-terminal 1–99 aa of Sec5) [12,13], and again washed 3 times with Washing buffer A. Then, beads were resuspended in Lysis buffer and divided into different microtubes (40 μl beads bed per microtube).

Each lysate was thawed and the desired protein amount was incubated for 45 min to 1 h with GST-Sec5-RBD-beads on the rotating wheel at 4°C. Beads were then quickly washed twice with 0.5 ml of Washing Buffer B (Tris.HCl 25 mM, NaCl 40 mM, MgCl2 30 mM, pH 7.5). Then 15 μl 2X Laemmli Sample Buffer were added to beads, which were then boiled 2 min, and immediately frozen or loaded on gel.

## Results and Discussion

### RGL2, RGL3, RalGDS and RalGPS2 are required for in vitro anchorage-dependent and anchorage-independent cell population growth

We started by evaluating the necessity of each of the six RalGEF for the *in vitro* proliferation and/or survival of four human NSCLC cell lines harboring different Ras mutations (A549, H23, H1299, H838), as compared to other cell lines where Ral and RalGEF proteins have been studied in the past: HeLa (cervix cancer), HEK-HT-HRas^G12V^ (transformed cell line, from now on named HekRasV12) and its isogenic pair HEK-HT (immortalized cell line, herein also named HekHT) [10]. Details on tissue histology and Ras mutation status of cell lines used in this work are given in Table 1.

Efficiencies of the siRNA oligonucleotides against the RalGEF (two to three siRNAs per gene) were confirmed by qPCR (S1 Fig): the target mRNA expression was below 30% of endogenous level for all siRNA sequences, at 72 h post-transfection, confirmed in at least two independent cell lines.

**Fig 1:**
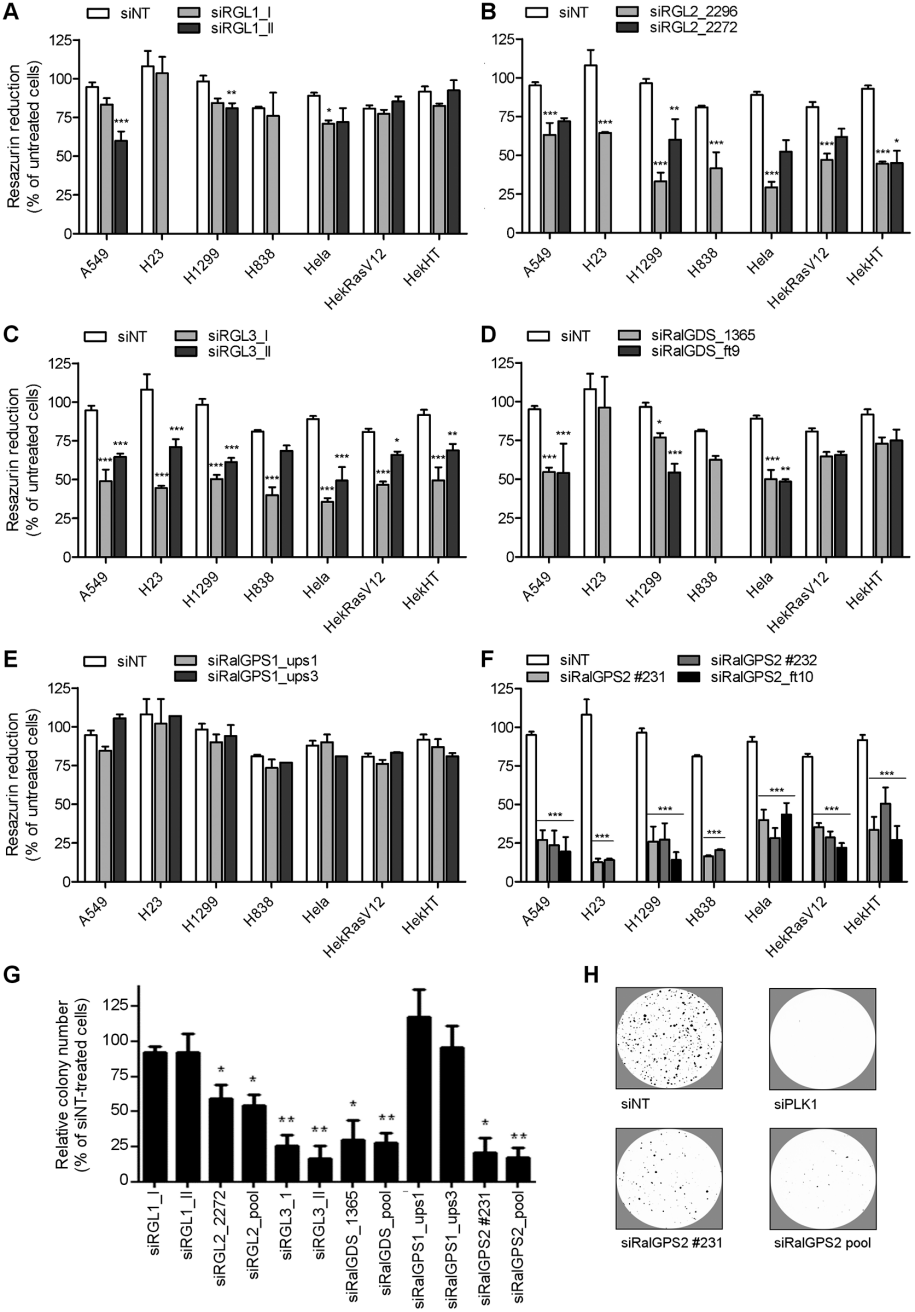
*RALGPS2* silencing induces pronounced anchorage-dependent and anchorage-independent cell population growth inhibition. (A—F) Cell population growth in adherence was measured using the resazurin reduction assay and is expressed as percentage of untreated (non-transfected) cells (mean ± SEM,). It was evaluated from 72 to 120 h after cell transfection, depending on the optimized condition for each individual cell line. Each panel depicts the effect of silencing one RalGEF with two or three independent siRNA oligonucleotides: (A) *RGL1*; (B) *RGL2*; (C) *RGL3*; (D) *RALGDS*; (E) *RALGPS1*; (F) *RALGPS2*. The effect of each siRNA was statistically compared with the effect obtained with siNT by Two-way ANOVA and Bonferroni posttests (*p ≤ 0.05, **p ≤ 0.01, ***p ≤ 0.001; *n* = 2 to 4 mean values from independent experiments, each done in quadruplicate wells). (G) Anchorage-independent growth of H1299 cells was evaluated over 14 days and is expressed as mean relative colony number with respect to siNT-treated cells. Data were collected from independent experiments where siPLK1 (positive control) originated none or very few colonies. (H) Illustrative images of H1299 colonies in agarose at 14 days of growth after siNT, siPLK1, siRalGPS2 #231, and siRalGPS2 pool, as indicated. Statistical comparison was done by One-way ANOVA with Dunnett’s posttest. (*p ≤ 0.05, **p ≤ 0.01, ***p ≤ 0.001; *n* = 2 (siRGL1 and siRALGPS1), 3 (siRalGPS1 and siRalGPS2) or 4 (siRGL2, siRGL3 and siRalGDS) independent experiments, each performed in triplicate wells.

The metabolic resazurin reduction assay was used to measure cell population growth. For each cell line, the transfection conditions and duration of the cell culture period were selected by assuring: 1- that cells did not reach confluence during the assay; 2- that the growth inhibitory effect of a non-targeting siRNA (siNT, the negative control) was minimal (< 15–20%) as compared with cells not submitted to transfection (untreated cells); and 3- that the growth inhibitory effect of a siRNA against a positive control, (we chose the polo-like kinase 1) was maximal (≥ 90%). Using this assay, we observed that *RGL1* and *RalGPS1* mRNA silencing had no detectable effect on cell population growth (Fig 1A and 1E, respectively), suggesting that they are dispensable for cell population growth, impacting substantially neither survival nor proliferation in these cells. However, the persistence of these two proteins due to high protein stability cannot be ruled out, since we could not follow protein decay over time due to unavailability of specific antibodies. Depletion of RGL2, RGL3, or RalGDS induced partial growth inhibition (varying from 12 to 70% as compared with siNT, Fig 1B, 1C and 1D). Interestingly, silencing of the Ras-independent RalGEF gene *RALGPS2* induced a very strong inhibition of cell population growth in all the NSCLC cell lines tested (up to 90%, Fig 1F). Furthermore, this effect was not specific of NSCLC cell lines, since it was observed also in cell lines with origin in kidney (HekHT/HekRrasV12) and cervix (HeLa cells), and in a not-transformed cell line (HekHT). The independence from the presence of an activated Ras gene might have been expected for a Ras-independent RalGEF.

Contrarily to immortalized cells, transformed cells display the ability to survive and proliferate in anchorage-independent growth conditions. The effect of RalGEF depletion in anchorage-independent growth was tested by scoring the number of colonies of H1299 cells in agarose jellified medium. While *RGL1* and *RALGPS1* silencing had no detectable effect, *RGL2*, *RGL3*, *RALGDS* and *RALGPS2* transient silencing substantially inhibited anchorage-independent growth of H1299 cells, paralleling the observations made in adherence conditions (Fig 1G). Interestingly, the inhibitory effect of *RGL3* and *RALGDS* silencing in anchorage independent growth was more pronounced (about 75% inhibition) than the effect observed on adherent cells (about 45% and 35% growth inhibition). Since *RALGPS2* silencing displayed the strongest phenotype, all further work focused on RalGPS2.

### RalGPS2 is required for in vitro cell survival

**Fig 2:**
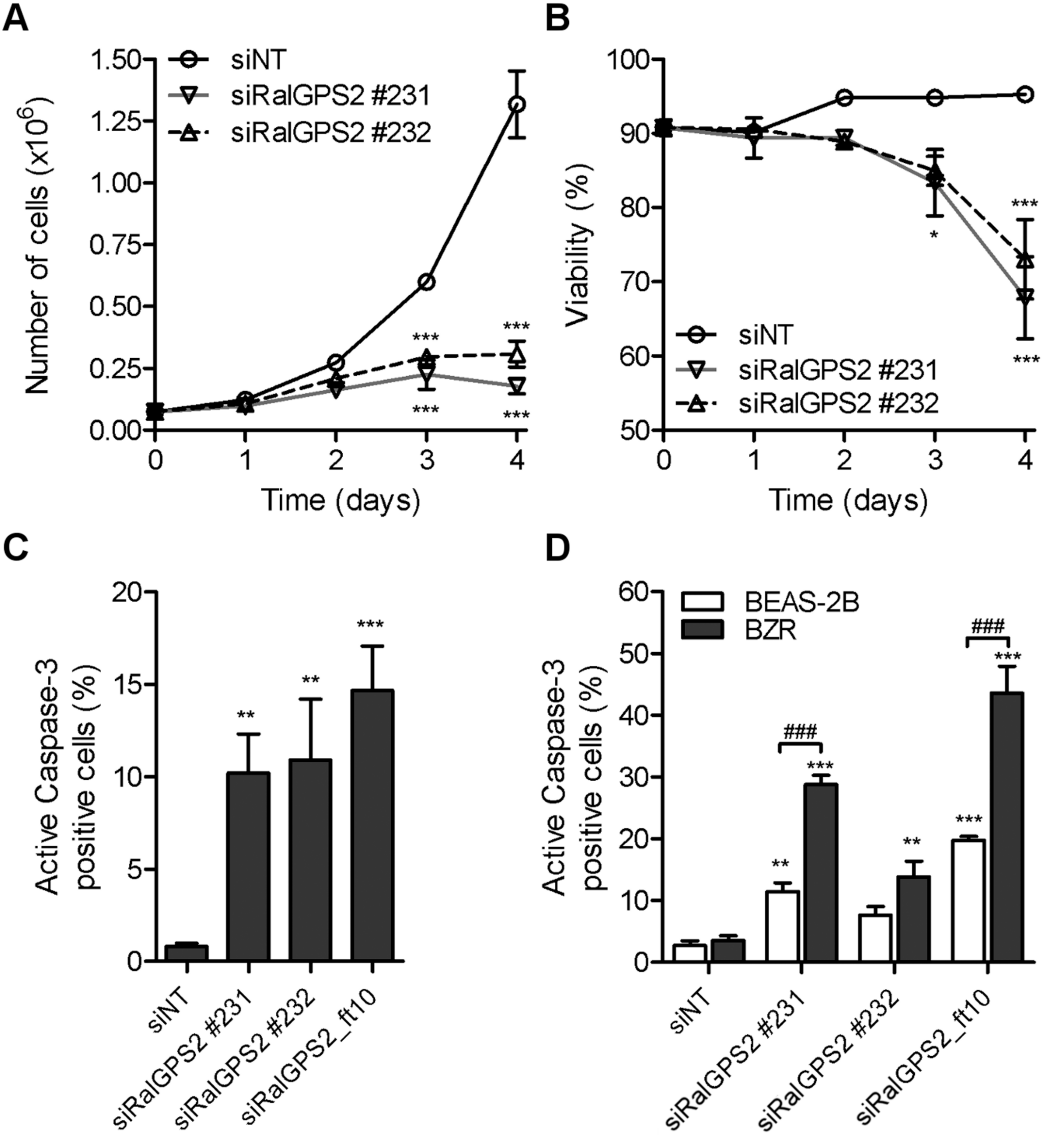
Cell survival is impaired by RalGPS2 depletion, especially in HRas^G12V^ transformed cells. (A) The number of viable H1299 cells and (B) the percentage of viability after induction of RalGPS2-depletion were obtained daily for four days with the Trypan Blue exclusion assay and statistically compared by Two-way ANOVA and Bonferroni posttests (*p ≤ 0.05, ***p ≤ 0.001; *n* = 2 independent experiments, each in triplicate). (C) The proportion of cells harbouring active Caspase-3 was evaluated 72 h post-transfection with siRalGPS2, by flow cytometry analysis, in H1299 cells and (D) in the isogenic pair of the immortalized BEAS-2B and HRas^G12V^-transformed BZR cells. Statistical significance was evaluated respectively by one-way ANOVA with Dunnett’s posttest, **p ≤ 0.01 and ***p ≤ 0.001, *n* = 4 or 5 independent experiments (C); and two-way ANOVA with Bonferroni posttest, ^###^ p ≤ 0.001, *n* = 2 or 3 independent experiments (D).

Upon depletion of RalGPS2 a high percentage of the cell population displayed phenotypic signs of cell death: cells rounding up, detaching and blebbing (S2 Fig). Furthermore, the Trypan Blue exclusion assay demonstrated that the strong growth inhibition at days 3 and 4 after transfection of H1299 cells (Fig 2A) was at least partially due to a 10 to 20% reduction of cell viability (Fig 2B). Therefore, RalGPS2 is required for cell survival. Moreover, cells were likely dying through apoptosis, as indicated by the 10 to 15% increase in the number of cells with activated Caspase-3 as analyzed by flow cytometry in H1299 cells (Fig 2C), and detection of cleaved Poly [ADP-ribose] polymerases (PARP) in Western Blots of A549 cells (S3 Fig). The RalGPS2 depletion-effect on apoptosis was further explored in a HRas^G12V^ oncogene context by silencing its expression in the isogenic pair of bronchial cell lines BEAS-2B (immortalized) and BZR (BEAS-2B expressing HRas^G12V^). Interestingly, Caspase-3 activation upon RalGPS2 depletion in BZR cells was about 2-fold more frequent than in the isogenic immortalized human bronchial epithelial cell BEAS-2B (Fig 2D). This result is suggestive of HRas^GV12^ oncogene-induced sensitization of cells to RalGPS2 for survival, although the global effect on cell population, as evaluated by resazurin reduction assay, was similar (S4 Fig), as for the other isogenic pair (HekHT and HekRasV12, Fig 1).

### RALGPS2-silencing effects go beyond RalA and RalB

**Fig 3:**
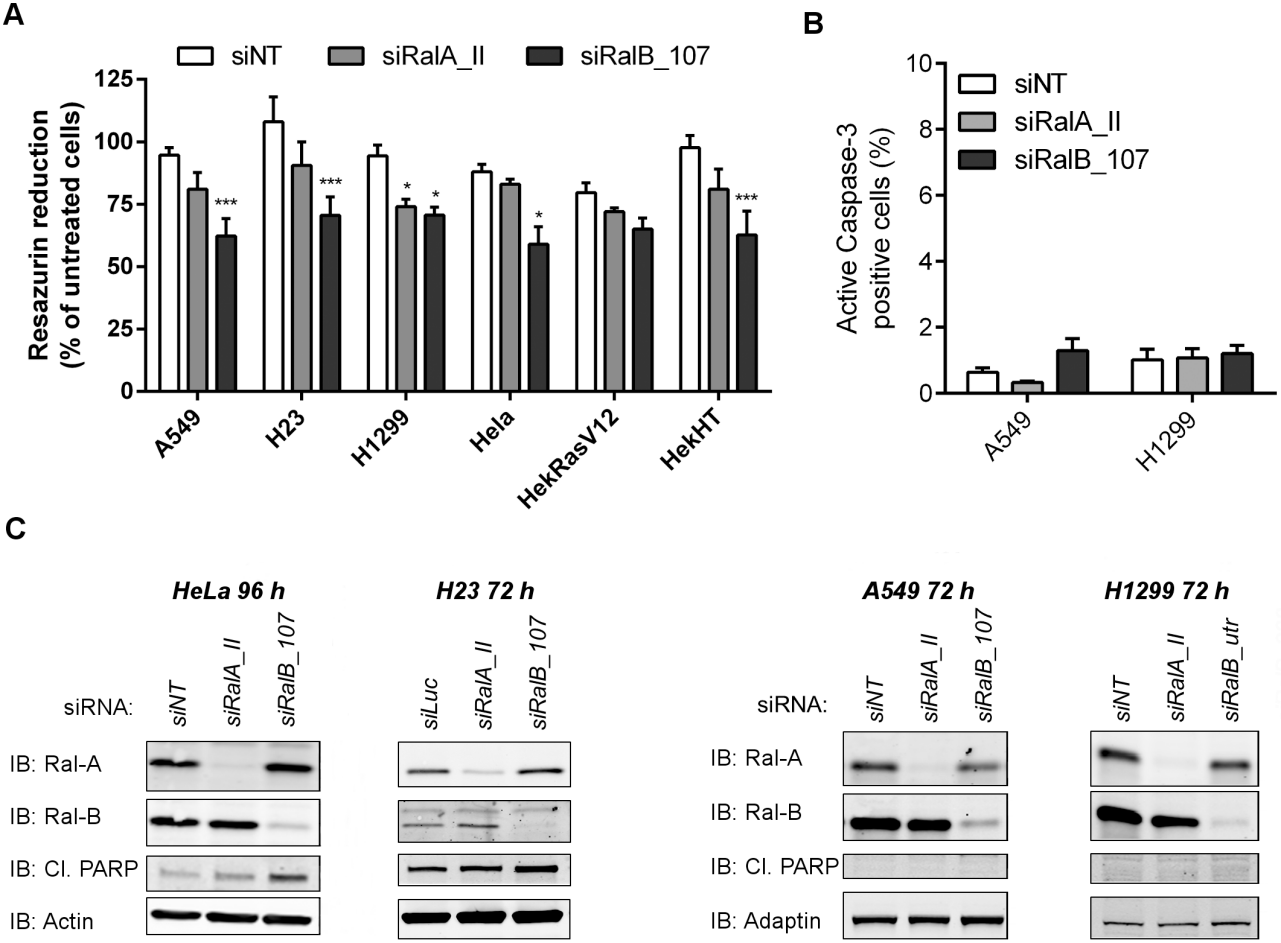
RalB, not RalA, is required for cell population growth, and survival does not explain it all. (A) Cell population growth was evaluated in the cell lines indicated in the graph by resazurin reduction 72–120 h post-transfection, and expressed as percentage of resazurin reduction relatively to untreated cells. The effect of each siRNA was statistically compared with the effect obtained with siNT (negative control) by Two-way ANOVA and Bonferroni posttests (*p ≤ 0.05, **p ≤ 0.01, ***p ≤ 0.001; *n* = 2 or 3 independent experiments, each the mean obtained from quadruplicate wells). (B) Caspase-3 activation was evaluated by flow cytometry analysis 72 h post-transfection and is expressed as the percentage of the cell population with positive staning. Statistical significance was evaluated by one-way ANOVA with Dunnett’s posttest but no significance was obtained (*n* = 3 independent experiments). (C) Western blots of the indicated cell lines and time points show RalA and RalB protein downregulation, cleaved Poly [ADP-ribose] polymerases (Cl. PARP) immunodetection and Actin or Adaptin loading control.

Next, we asked to what extent Ral proteins are implicated downstream RalGPS2 in cell population growth and survival. *RALA* silencing had no effect on cell population growth (except for a partial 20% growth inhibition in only one cell line, H1299, out of the six tested), while upon RalB depletion there was a partial inhibition of growth in five out of six cell lines tested (24 to 38% mean difference from siNT, Fig 3A), although less substantial than what was observed by silencing *RALGPS2* in the same cells (61 to 96% mean difference from siNT in lung cancer cell lines, 41 to 62% mean difference from siNT in non-lung cancer cell lines, Fig 1F). Furthermore, in contrast to the established role of RalB in cell survival [14], we did not observe apoptosis upon RalB depletion in neither A549 nor H1299 lung cell lines, as analyzed by Caspase-3 activation (Fig 3B) and cleaved PARP (Fig 3C). These results indicated that the population growth inhibition induced by *RALB* silencing was not due to cell death, at least in these two NSCLC cell lines, and raised the possibility that *RALB* silencing might affect cell cycle progression, as addressed in the following section.

Recent Theodorescu’s lab work showed that in lung cancer cell lines the effect of silencing *RALA* and/or *RALB* is cell line dependent, and likely dependent on the type of Ras mutation present [7]. Consistently with this cell-type variability, we could observe a moderate increase in cleaved PARP on Western blots with HeLa and lung cancer H23 cell lines, but not with A549 and H1299 cell lines (Fig 3C), suggesting that cell death might indeed contribute to RalB depletion effects in some cell types.

Furthermore, we observed that siRNA transfection conditions affect the apoptosis readout, raising the usually overlooked issue of interaction of transfection agents with the treatments to which the effects are attributed. *RALB* silencing did not induce apoptosis in the low-toxicity siRNA transfection conditions used in this work (see M&M for details), but under harsher conditions (two sequential reagents for double siRNA/DNA transfections) it was possible to detect cleaved PARP on Western blots of H1299 cells (S5 Fig), suggesting that apoptosis induction by RalB depletion might be dependent on the existence of sufficient stress insult to cells.

**Fig 4:**
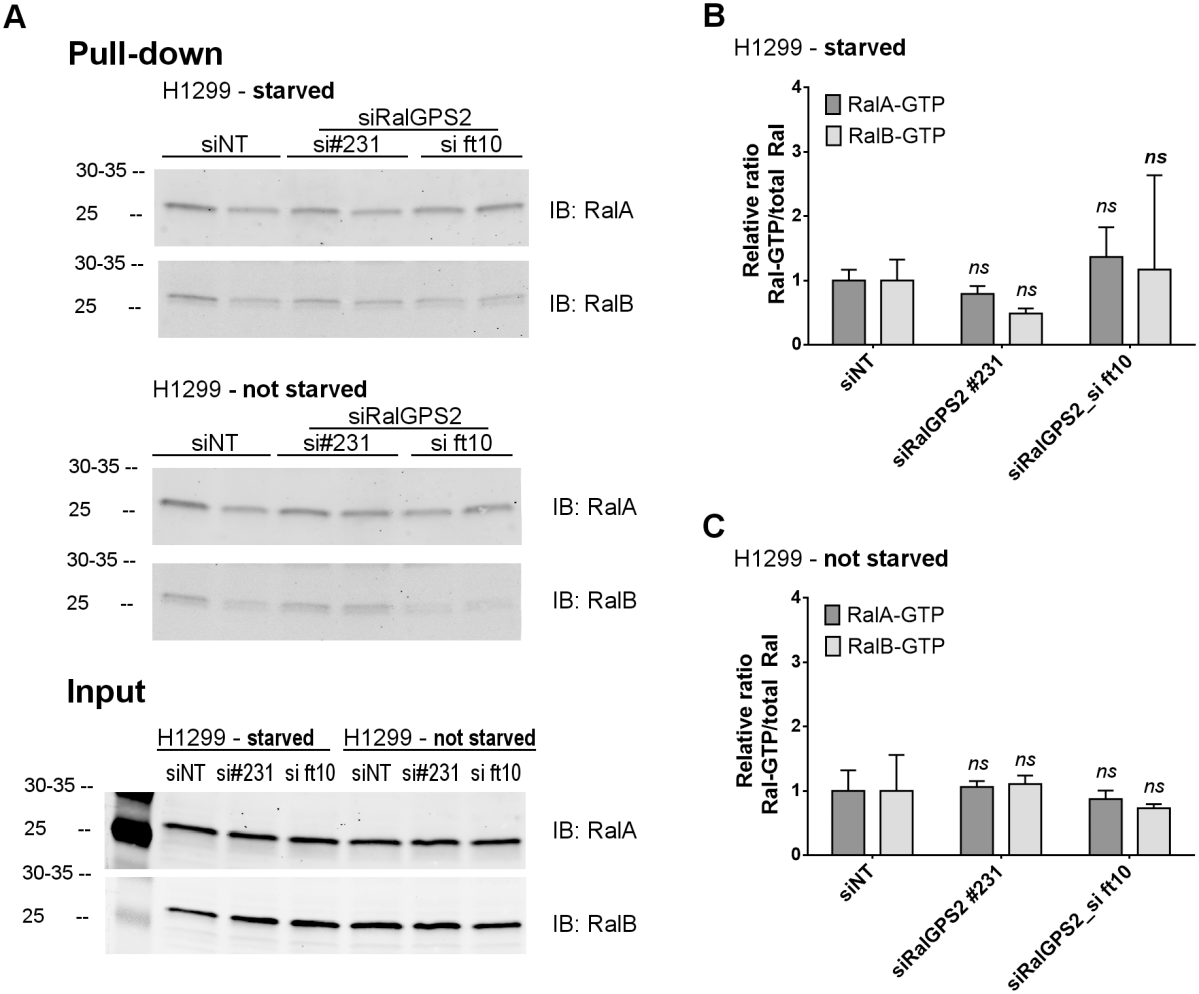
Ral-GTP levels in H1299 cells after RalGPS2 depletion. H1299 “starved” cells were obtained by overnight (~19 h) culture without serum before lysis at 48 h post-transfection. (A) Pull-down was performed as independent duplicates from the same lysates at 0.4 mg protein/pull-down/lane (0.5 mg/ml). The input corresponds to 25-fold less protein (16 μg/lane). 20 μg GST-Sec5-RBD and 40 μl beads were used per pull-down. (B) Quantification of immunoblots (IB) Ral-GTP/total Ral ratios from “starved” cells and expressed relatively to siNT (mean ± SD, *n* = 2 or, in the case of siRalGPS2_ft10, 3 independent pull-downs). (C) IB signal quantification from the experiment with cells “not starved” (mean ± SD, *n* = 2 independent pull-downs). No significant differences were found among means at a significance level of 0.05 (Two-way ANOVA with Bonferroni posttests).

Additionally, we performed standard pull-down assays to assess the impact of RalGPS2 depletion on GTP-bound RalA and RalB levels (Fig 4 and S6 Fig). Generally, the Ral-GTP levels of NSCLC cells (H1299, A549, H358) were constitutively very low, as compared to BEAS-2B (immortalized human bronchial cells) or BT-549 (breast cancer cells) (S6A Fig). This could be due, at least partially, to high RalGAP activity. Indeed, we could substantially increase RalA-GTP and RalB-GTP levels upon *RALGAPB* subunit silencing with 3 out 4 independent siRNAs in H1299 cells (S6B–S6D Fig).

In H1299 cells, upon depletion of RalGPS2 with 2 independent siRNAs, there was no consistent perturbation of the already faint Ral-GTP signals, either in cells cultured in the presence of FBS or in cells starved overnight (Fig 4). These data are consistent with the conclusion that the effects of RalGPS2 depletion are Ral-independent, since no impact could be detect on Ral GTP-loading, at least at global level.

### RalGPS2 and RalB regulate cell cycle progression

**Fig 5:**
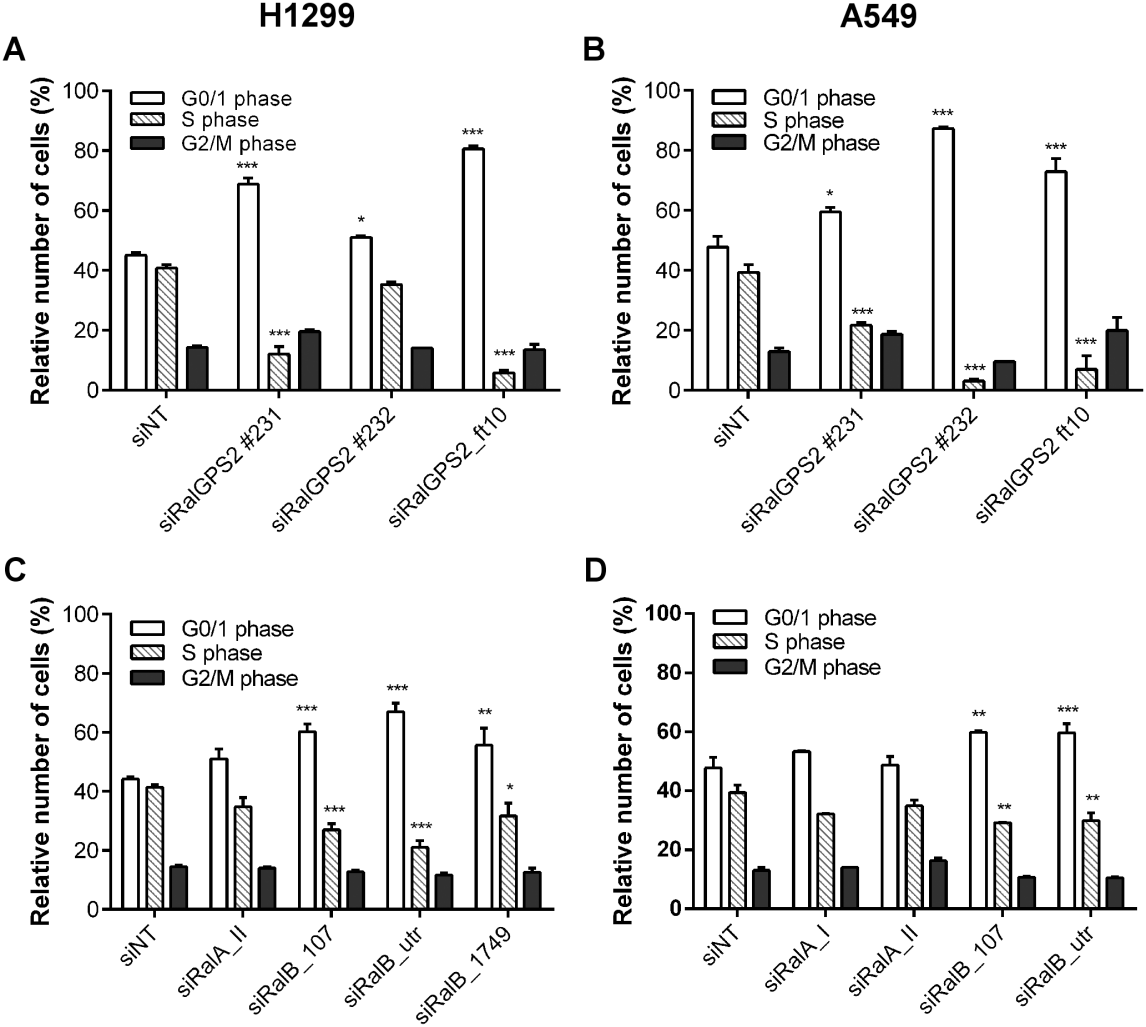
*RALGPS2* or *RALB* silencing cause G0/G1 cell cycle arrest. Flow cytometry analysis of cell cycle distribution was performed in singlet events negative for active Caspase-3, 72 h post-transfection of (A and C) H1229 and (B and D) A549 cells. The effect of each siRNA was statistically compared with the effect obtained with the control siRNA siNT by Two-way ANOVA and Bonferroni posttests (*p ≤ 0.05, **p ≤ 0.01, ***p ≤ 0.001, *n* = 3 or 4 independent experiments).

Next, we investigated a possible role of RalB and RalGPS2 in cell cycle progression. *RALGPS2* silencing induced a strong reduction of the percentage of cells in S-phase, reaching as low as 3%, and increased the percentage of cells in the G0/G1-phase in both H1299 and A549 cells from around 45–48% up to 81 and 87%, respectively (Fig 5A and 5B). Very likely, this is not related with the previously described cytokinesis function of RalGPS2 [4], which might lead to a small increase in the M population. In fact, there was no significant change detected in the G2/M fraction.

However, it should be added that in the immortalized BEAS-2B, *RALGPS2* silencing induced an increase in the number of polyploid cells instead of a G1-arrest (S7 Fig). This means that RalGPS2 depletion may have different phenotypic defects on cell cycle depending on cell type.

Interestingly, *RALB* silencing, but not *RALA* silencing, had a mild effect on cell cycle progression in both H1299 (Fig 5C) and A549 (Fig 5D) cells, although even less substantially in the latter. Simillar results were obtained when silencing both *RALA* and *RALB* together as compared to silencing *RALB* only (S8 Fig).

### RalGPS2 depletion, but not RalB depletion, is associated with p21 & p27 upregulation and with Skp2 downregulation

**Fig 6:**
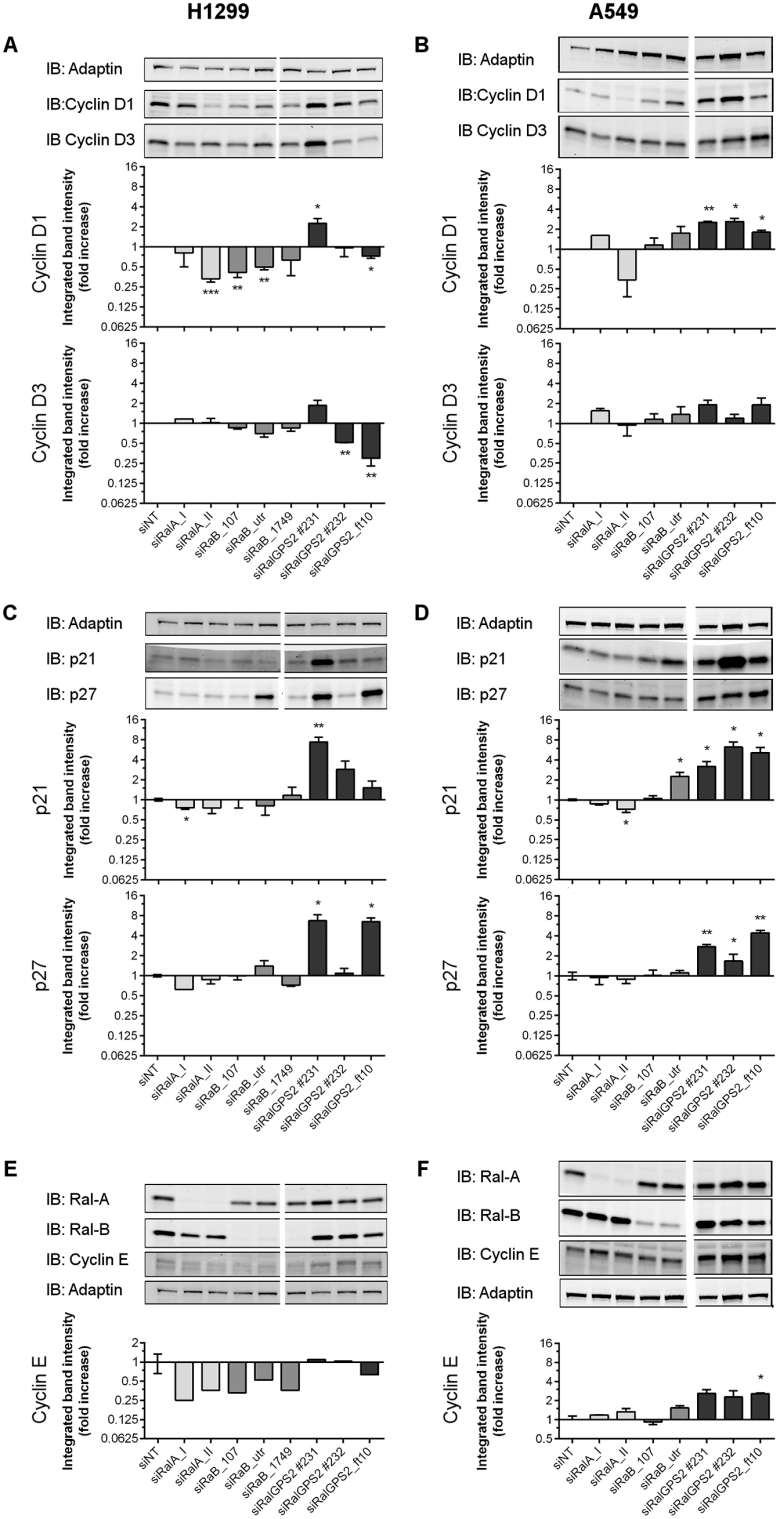
RalGPS2 depletion increases p21 and p27 protein expression. (A and B) Representative Western blots and quantification (mean ± SEM) of up to 4 independent experiments of the expression of Cyclin D1 and Cyclin D3, and (C and D) of the Cyclin dependent kinase inhibitors p21 and p27. (E and F) RalA and RalB protein downregulation efficiency is shown together with Cyclin E expression and its respective quantification data (mean ± SEM) corresponding to 1 and 2 independent experiments in H1299 and A459, respectively. Data were collected 72 h after siRNA transfection. Light-grey bars—siRalA, medium-grey bars—siRalB, and dark-grey bars—siRalGPS2. Statistics: the difference from the reference (theoretical value 1) was evaluated by One sample t test, two-tailed, *p ≤ 0.05, **p ≤ 0.01. The vertical white lines in the Western blots indicate positions were gel images were cut in order to juxtapose non-adjacent lanes coming from the same gel.

In order to clarify the mechanisms underlying the regulation of cell cycle by RalGPS2 and RalB, we first tested whether Ral and RalGPS2 depletion could impact Cyclin D1 and D3 levels (the Cyclin D isoforms we could detect in both H1299 and A549 cells). In H1299 cells, RalB depletion consistentely led to reduced Cyclin D1 levels, (Fig 6A). However, this was not observed in A549 cells (Fig 6B). Previous reports connected Ral and Cyclin D1 expression through NF-kB [15], and RalB was linked to TBK1/NF-kB signaling [16,17]. Therefore it can be hypothesized that RalB depletion effect in Cyclin D1 expression and cell cycle regulation in H1299 cells is mediated by TBK1/NF-kB signaling, but this was not further investigated. However, in contrast to RalB depletion in H1299 cells, RalGPS2 depletion did not impact coherentely Cyclin D expression (Fig 6A and 6B).

The impact of *RAL* and *RALGPS2* silencing on the Cyclin dependent kinase inhibitors p21 and p27 was also evaluated. In contrast to the depletion of RalA or RalB, RalGPS2 depletion led to increased levels of p27 in both H1299 and A549 cells. P21 also significantly increased, at least in A549 cells, while in H1299 the increase reached statistical significance only with one of the siRNA oligonucleotides (Fig 6C and 6D). Overall, p21 and p27 results are consistent for p21/27 to be the main mediators of the cell cycle impact of *RALGPS2* silencing. Since this was not mimicked by silencing none of the *RAL*, and total Ral-GTP levels in H1299 cell are not dependent on RalGPS2 (Fig 4), RalGPS2 appears to work in cell cycle independently of these two GTPases. This is not the first time that a RalGEF is suggested to have functions beyond Ral regulation. Chaning Der´s and co-workers claimed the same for RGL2 [18] and for RalGDS regulation of AKT kinase activation [19].

Another cell cycle regulator implicated in G1/S transition is Cyclin E, which is absent during early G1 and peaks at the G1/S phase transition, where it helps to assure that p27 is degraded (see [20] for a review). Therefore, Cyclin E expression can help to distinguish between early and late G1 phases. In the A549 cell line Cyclin E expression was augmented in RalGPS2-depleted cells as compared with control or with Ral-depleted cells (Fig 6F). Averaging the effect of the 3 independent siRNA against *RALGPS2*, the increase of Cyclin E was statisticaly significant (p = 0.0045, One-sample t-test, *n* = 3). This suggests that upon *RALGPS2* silencing A549 cells are arrested at late G1. In H1299 cells having quantifyable blots was very difficult due to faint Cyclin E detection in this cell line (Fig 6E). However, by averaging the effect of the independent siRNA in the shown blot, the decrease in Cyclin E expression with siRalB was statistically significant (*p* = 0.0013, One sample t-test, *n* = 4; with siRalA *p* = 0.0503, *n* = 2), suggesting that upon *RALB* silencing H1299 cells are arrested at early G1.

**Fig 7:**
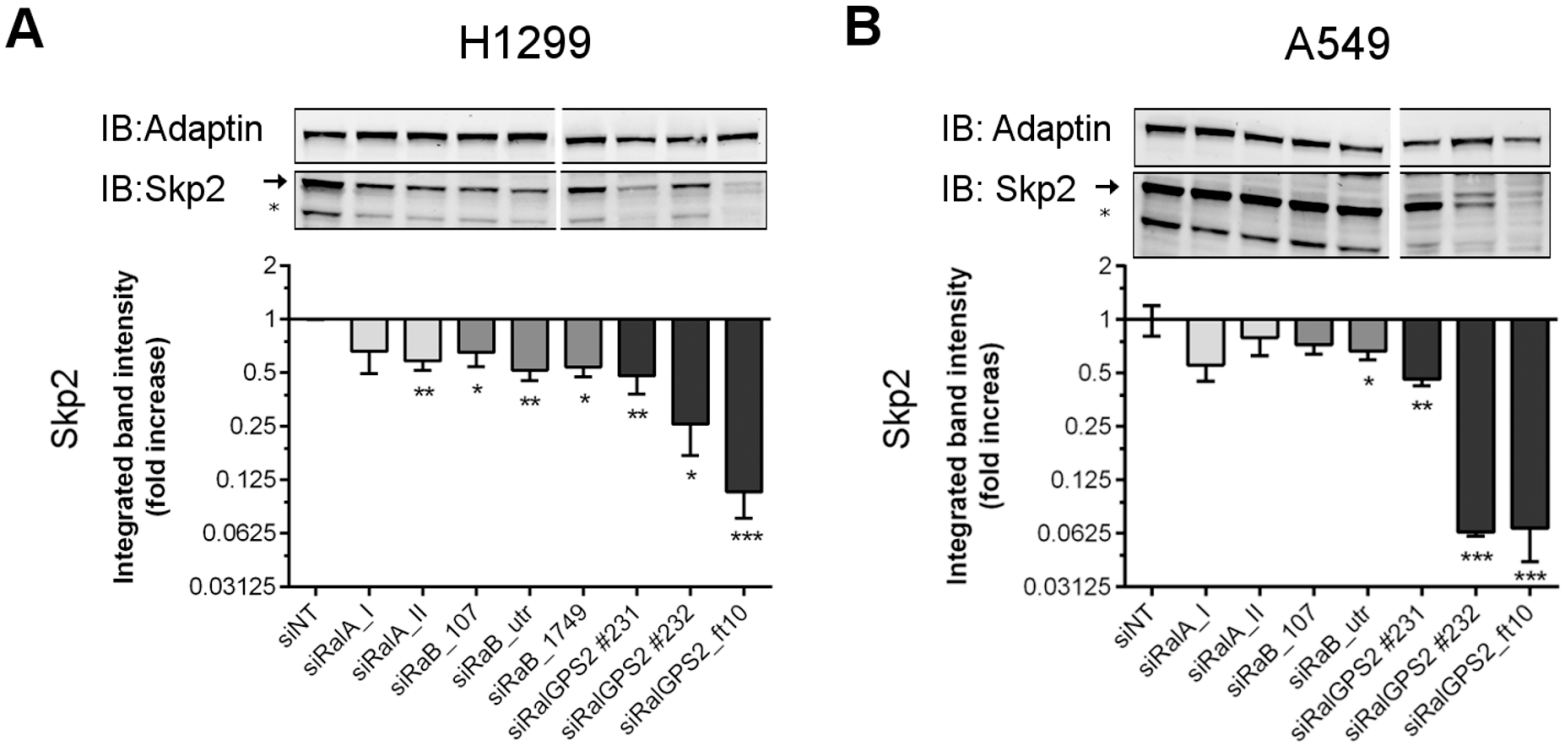
Skp2 protein levels decrease upon RalGPS2 depletion. Representative Western blots and respective quantification (mean ± SEM) of 3–5 independent experiments of the expression of Skp2 (upper band, arrow) in H1299 (A) and A549 (B) cells, 72 h after siRNA transfection. The Skp2 faster migrating band (*), at the level of the 40 KD standard, has not been described so far but accompanied the variation of the upper band. Light-grey bars—siRalA, medium-grey bars—siRalB, and dark-grey bars—siRalGPS2. Statistics: the difference from the theoretical reference value 1 was evaluated by One-sample t-test, two-tailed, *p ≤ 0.05, **p ≤ 0.01, ***p ≤ 0.001.

In addition, we evaluated the levels of a common post translational regulator of both p27 and p21, S-phase kinase-associated protein 2 (Skp2), the target-recognition protein in the Skp2, Cullin, F-box containing (SCF^Skp2^) complex, which is a multi-protein E3 Ubiquitin Protein Ligase that targets p27 and p21 to proteasomal degradation from the G1-S phase transition to the completion of M-phase of cell cycle [21]. Strikingly, there was a strong and consistent reduction in Skp2 protein levels upon RalGPS2 depletion in both H1299 and A549 cells (Fig 7A and 7B, respectively).

Silencing of *RALA* or *RALB* in H1299 cells also reduced Skp2 expression but much less substancially (Fig 7A), and this can be a consequence of early G1-arrested cells, since Skp2 levels are kept low in early G1 by the Adenomatous Polyposis Coli protein [22]. Furthermore, we were able to determine that Skp2 downregulation uppon RalGPS2 depletion occured also at the mRNA level, suggesting a transcriptional or a mRNA stablity mechanism (S9 Fig). Also, siRalGPS2 #232 induced a lower *SKP2* mRNA down-regulation than the 2 other siRalGPS2 (S9 Fig) and produced no p27 up regulation (Fig 6C), possibly because of its lower efficiency in silencing *RALGPS2* in H1299 cells (18% *vs*. 7%, S1 Fig).

Very interestingly, there is a striking similarity between the RalGPS2 depletion effects that we observed and those described upon Skp2 knockdown in NSCLC cancer cells: suppression of DNA synthesis and apoptosis [23], increased p27 protein level and reduced cell growth [24], or elevated p27 protein level, decreased cell proliferation, and apoptosis (including in A549) [25]. These observations taken together support the model that RalGPS2 controls cell survival and growth, independently of Ral, via a mechanism involving the regulation of Skp2.

## Conclusion

Unexpectedly, when silencing RalGEF genes in lung cancer cells none of the Ras-dependant RalGEF stood out but one of the Ras-independent RalGEF did: *RALGPS2* silencing produced the strongest inhibition of cell population growth among all six RalGEF in four NSCLC cell lines. At least in H1299, BZR and BEAS-2B cells, *RALGPS2* silencing was associated with a decrease in cell survival. In addition, at least in H1299 and A549 cell lines, *RALGPS2* silencing was associated with a G0/G1 phase cell cycle arrest, in the case of A549 at late G1. Furthermore, although a novel cell cycle regulation function of RalB was unraveled in H1299 cells, the molecular effects of RalGPS2 depletion on the Cyclin inhibitors p27 and p21 were not phenocopied by depletion of either RalA, RalB or both, in both H1299 and A549 cells, neither was Ral activation influenced by RalGPS2 depletion. Interestingly, RalGPS2 depletion is associated with downregulation of an important E3 Ubiquitin Protein Ligase, the Skp2, which may explain the up-regulation of the cell cycle inhibitors p21 and p27. Given the important role of Skp2, p21 and p27 regulation for cancer cell proliferation, survival and invasion [26–28], future research on the molecular connection between Skp2 regulation and RalGPS2 is warranted for new avenues of intervention. It is also possible that other unforeseen RalGPS2 targets may exist, and therefore the RalGPS2 Ral-independent signaling pathways deserve careful attention in the future.

## Supporting Information

S1 Fig Target mRNA knockdown with the different anti-RalGEF siRNA. Results are shown for 2 cell lines for the majority of siRNA sequences. *n* = 1, 2 or 3 independent experiments in each cell line. Each quantification was performed in triplicate. In H1299 cells mRNA mean values obtained with siRNARalGPS2 #231 and siRalGPS2 #232 are significantly different (*p =* 0.0237, unpaired t-test).
(TIF)

S2 Fig Appearance of H1299 cells at 48 and 96 hours after transfection with the indicated siRNA. (TIF)

S3 Fig Cleaved PARP immunodetection in Western blots. Total protein lysates of A549 cell line were obtained 72 h post-transfection at 12 nM siRNA. A control sample was loaded at decreasing relative volumes to confirm that Adaptin detection was not saturated. Quantification of Cl. (cleaved) PARP normalized by Adaptin signal is also shown under the bands. *siRalB_333 targets sequence 5'-CUGACAGUUAUAGAAAGAAA-3').
(TIF)

S4 Fig Relative cell population growth of BEAS-2B and BZR cells depleted of RalA, RalB or RalGPS2. SiRNA were at 12 nM and medium contained BSA at 0.2%.The resazurin reduction assay was performed 72 h post-transfection. Data are means ± SD of 2 to 4 independent experiments, each performed in quadruplicate wells.
(TIF)

S5 Fig Cleaved PARP detection in H1299 cells after siRalB and plasmid double transfection. Western blot lanes 1 to 3 are of protein samples of siRNA-transfected only, and lanes 4 and 5 are of siRNA and plasmid double-transfected H1299 cells. The vertical white lines in the Western blots indicate positions were gel images were cut in order to juxtapose non-adjacent lanes coming from the same gel. siRNA transfection were performed as described under M&M in 6-well plate. Past 24 h, 3 μl of jetPRIME transfection reagent (Polyplus-transfection SA, France) were used for a total of 2 μg control empty plasmid/well. Samples were collected 72 h post-initial siRNA transfection.
(TIF)

S6 Fig Low Ral-GTP levels in H1299 and its up-regulation by RalGAP depletion. (A) Comparison of active GTP-loaded RalA and RalB in the indicated cell lines. (B) In H1299 cells, impact on GTP-loading of RalA and RalB upon silencing of the beta subunit (*RALGAPB*) of the two RalGAP complexes with four independent siRNA (ft5, ft6, ft7, ft8) from Qiagen (FlexiTube siRNA for human *RALGAPB* SI04288669 CCCTCGGAGACCAAAGGTTAA; SI04278337 ACCCGTGAATAGATTAAGTAT; SI04201057 GAACGCTATGATGATATAGAA; SI04136748 CACCGACATTGTCAACAAGTA). “GTP” indicates in vitro GTP-loading control that was performed before pull-down by adding to control lysate samples 1/10^th^ volume of Loading Buffer (150 mM EDTA solution), then 1/100^th^ volume GTPγS (200 μM final concentration), and incubating at 30°C for 15 min with gentle rotation or agitation. The reaction was stopped by transferring the tube to ice and adding 1/10th volume of STOP Buffer (600 mM MgCl_2_ solution). (C) Quantifications from panel B. (D) RALGAPB knockdown efficiency measured by qPCR (Taqman probes Hs00384265_m1).
(TIF)

S7 Fig DNA content histograms of RalGPS2 depleted BEAS-2B cells. Graphics #1 and #2 correspond to 2 independent experiments. Samples were collected 72 h after siRNA transfection with 12 nm siRNA (1 μl Lipofectamine RNAiMAX: 12 pmol siRNA). FL3-A, Fluorescence channel 3-area of the peak of the signal.
(TIF)

S8 Fig Double siRalA and siRalB transfection of A549 cells. (A) Cell cycle distribution of double transfection were not different from siRalB single transfections. (B) Western blots of the respected double transfected cells show efficient RalA and RalB depletion.
(TIF)

S9 Fig *SKP2* mRNA levels in H1299 cells depleted of RalGPS2. Quantitative real-time RT-PCR was performed in samples collected 72 h after transfection. Data is mean ± SEM of 2 or 3 (siRalGPS2_ft10) independent experiments, each quantified in triplicate. Significance of mean differences was evaluated with one-way ANOVA and Dunnett’s post-test, ***p ≤ 0.001.
(TIF)
