# Supplementary figures and images for: RalGPS2 Is Essential for Survival and Cell Cycle Progression of Lung Cancer Cells Independently of Its Established Substrates Ral GTPases
Source: PLoS One. 2016 May 5;11(5):e0154840. doi: 10.1371/journal.pone.0154840 (PMC4858283; doi:10.1371/journal.pone.0154840)

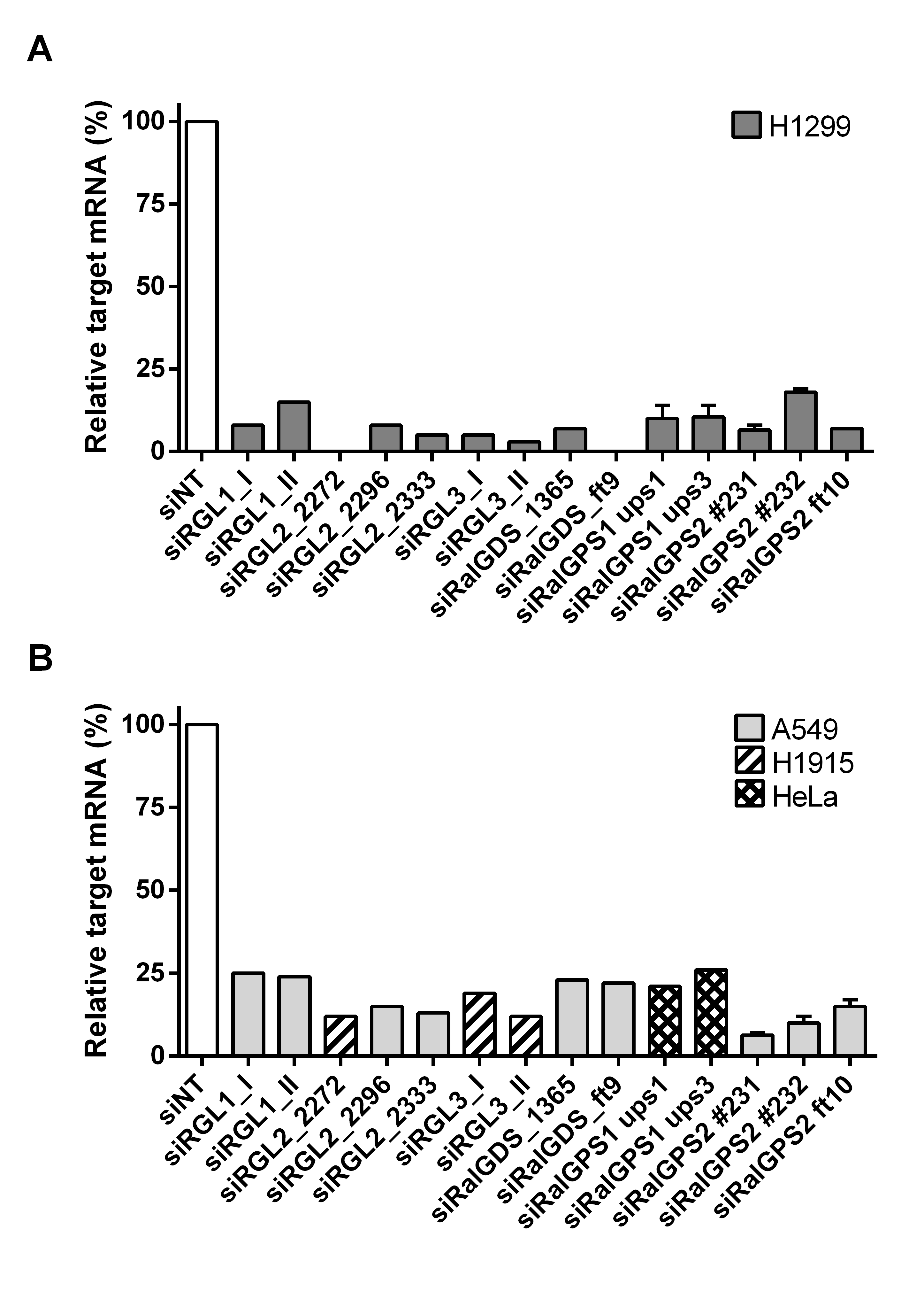

Supplement: S1 Fig — Results are shown for 2 cell lines for the majority of siRNA sequences. n = 1, 2 or 3 independent experiments in each cell line. Each quantification was performed in triplicate. In H1299 cells mRNA mean values obtained with siRNARalGPS2 #231 and siRalGPS2 #232 are significantly different (p = 0.0237, unpaired t-test). (TIF) [file pone.0154840.s001.tif]

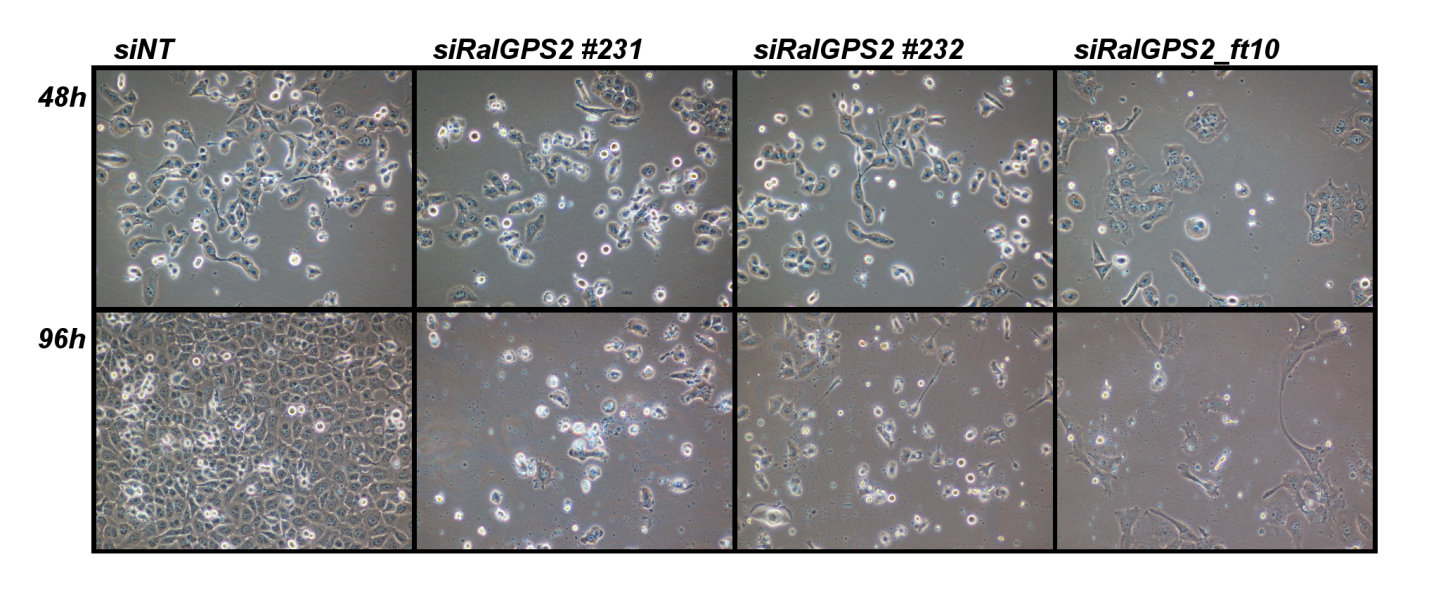

Supplement: S2 Fig — (TIF) [file pone.0154840.s002.tif]

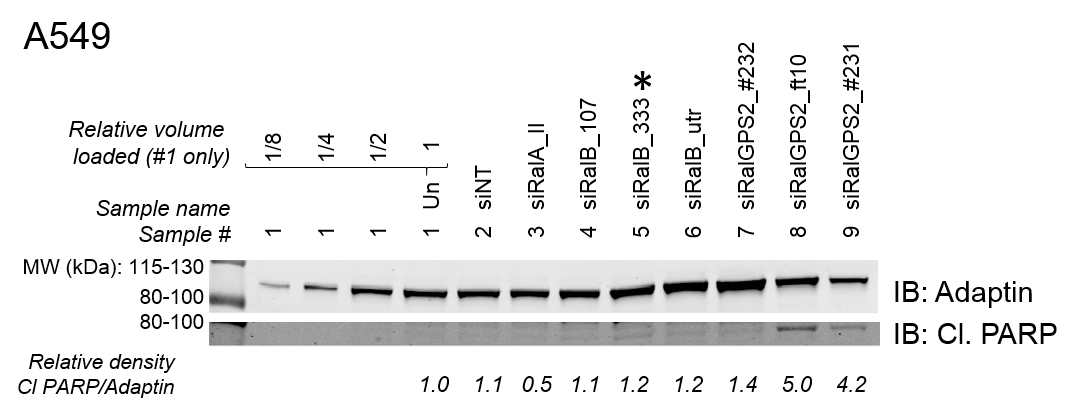

Supplement: S3 Fig — Total protein lysates of A549 cell line were obtained 72 h post-transfection at 12 nM siRNA. A control sample was loaded at decreasing relative volumes to confirm that Adaptin detection was not saturated. Quantification of Cl. (cleaved) PARP normalized by Adaptin signal is also shown under the bands. *siRalB_333 targets sequence 5'-CUGACAGUUAUAGAAAGAAA-3'). (TIF) [file pone.0154840.s003.tif]

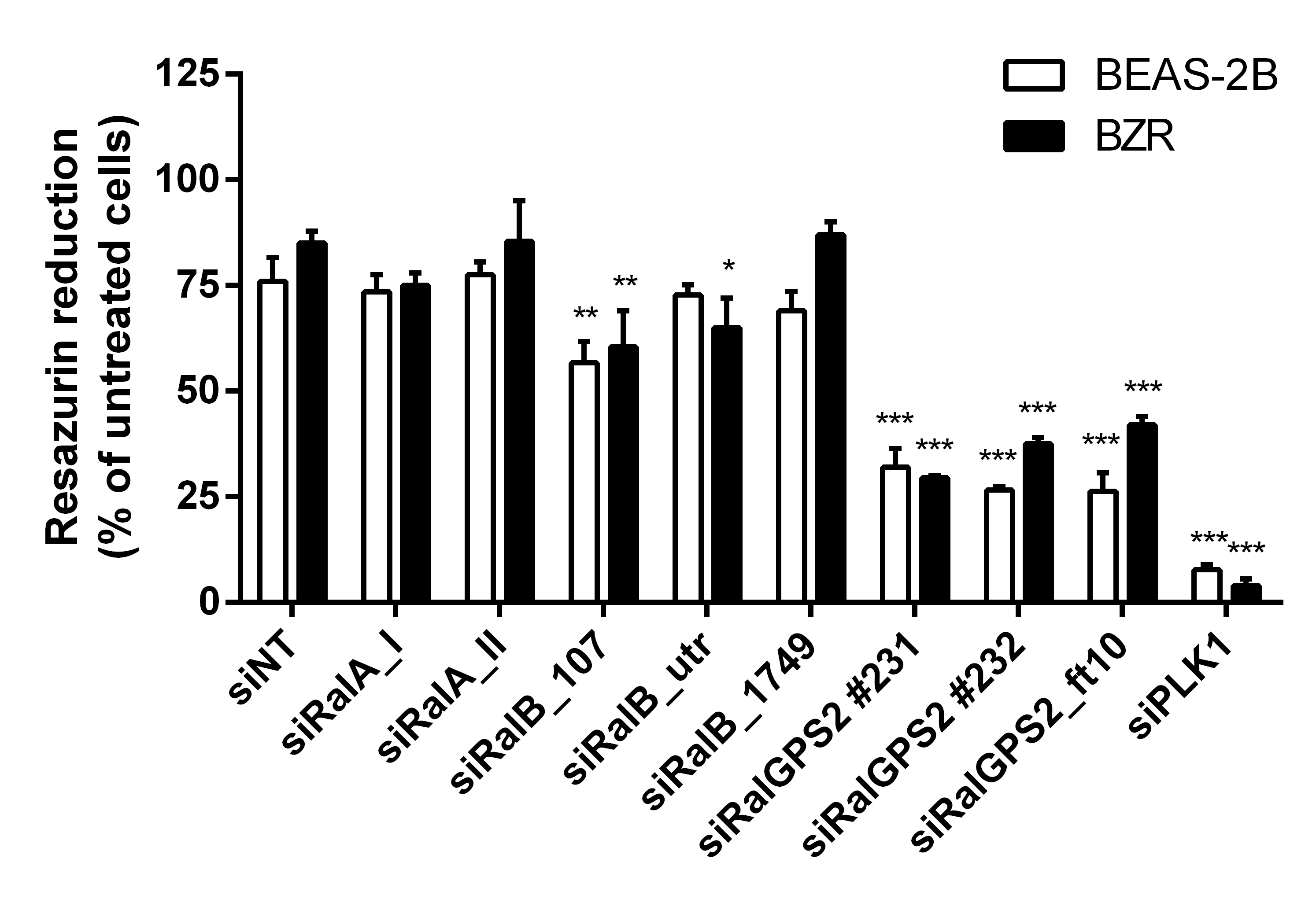

Supplement: S4 Fig — SiRNA were at 12 nM and medium contained BSA at 0.2%.The resazurin reduction assay was performed 72 h post-transfection. Data are means ± SD of 2 to 4 independent experiments, each performed in quadruplicate wells. (TIF) [file pone.0154840.s004.tif]

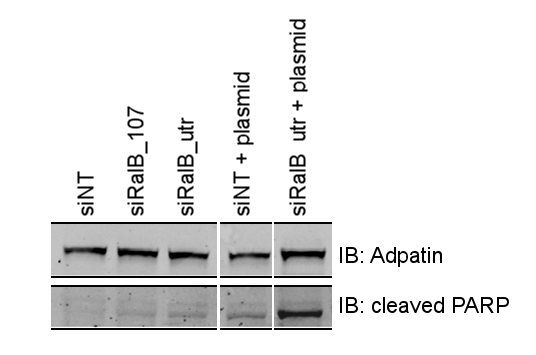

Supplement: S5 Fig — Western blot lanes 1 to 3 are of protein samples of siRNA-transfected only, and lanes 4 and 5 are of siRNA and plasmid double-transfected H1299 cells. The vertical white lines in the Western blots indicate positions were gel images were cut in order to juxtapose non-adjacent lanes coming from the same gel. siRNA transfection were performed as described under M&M in 6-well plate. Past 24 h, 3 μl of jetPRIME transfection reagent (Polyplus-transfection SA, France) were used for a total of 2 μg control empty plasmid/well. Samples were collected 72 h post-initial siRNA transfection. (TIF) [file pone.0154840.s005.tif]

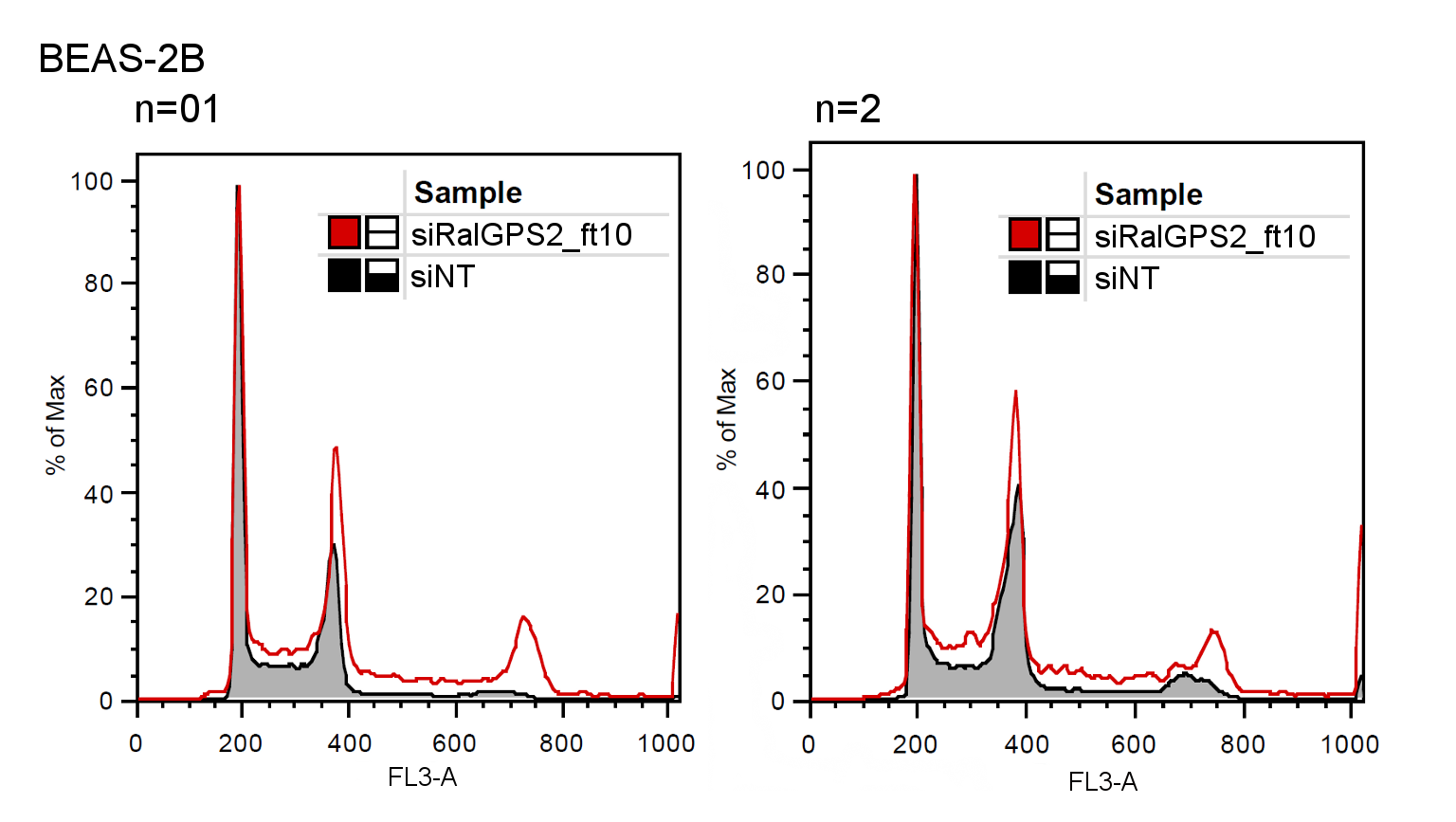

Supplement: S7 Fig — Graphics #1 and #2 correspond to 2 independent experiments. Samples were collected 72 h after siRNA transfection with 12 nm siRNA (1 μl Lipofectamine RNAiMAX: 12 pmol siRNA). FL3-A, Fluorescence channel 3-area of the peak of the signal. (TIF) [file pone.0154840.s007.tif]
